# COGNITIVE PERFORMANCE DIFFERENTIALLY MODERATES THE ASSOCIATION BETWEEN GAIT VELOCITY AND FALL RISK

**DOI:** 10.1093/geroni/igae098.2711

**Published:** 2024-12-31

**Authors:** Sandra Hundza, Stuart MacDonald, Marc Klimstra, Markus von Hacht

**Affiliations:** University of Victoria, Victoria, British Columbia, Canada; University of Victoria, Victoria, British Columbia, Canada; University of Victoria, Victoria, British Columbia, Canada; University of Victoria, Victoria, British Columbia, Canada

## Abstract

Cognitive-walking dual-task paradigms are an effective tool for assessing fall risk. Surprisingly, the moderating influence of cognitive performance on the association between gait velocity (GV) and fall risk has not been explored. The present investigation examines whether cognitive performance moderates the relationship between GV and fall risk. Community-dwelling older adults (76 years ±3.44) were classified as fallers and non-fallers based on self-report (at least one fall in the past 12 months). GV was indexed using the GAITRite system while counting backwards by serial 7s. Performance on the serial 7s task was recorded, with Adobe Audition subsequently employed to index cognitive function in terms of the number of counts (NC) and percentage of true counts (PTC) from the recorded audio files. Logistic regression was employed to examine the predictive influence of GV on the likelihood of fall risk classification (GV model), as well as the moderating influence of NC and PTC on the GV-fall risk association (GCI model). Notably, there was a significant moderating effect of PTC on the GV-fall risk association for individuals with PTC scores ≥-2.41 units (Johnson-Neyman analysis); those with lower GV were at increased risk of falling, with this relationship magnified as PTC scores increased. The sensitivity of the GCI interaction model (88%) represented a 17% improvement over the GV model. This investigation is the first to demonstrate the importance of interactions between gait and cognition measures in fall risk modelling, and underscores the potential clinical utility of including cognitive performance measures in dual-task paradigms.

